# Bacterial Colonization of Host Cells in the Absence of Cholesterol

**DOI:** 10.1371/journal.ppat.1003107

**Published:** 2013-01-24

**Authors:** Stacey D. Gilk, Diane C. Cockrell, Courtney Luterbach, Bryan Hansen, Leigh A. Knodler, J. Antonio Ibarra, Olivia Steele-Mortimer, Robert A. Heinzen

**Affiliations:** 1 *Coxiella* Pathogenesis Section, Rocky Mountain Laboratories, National Institute of Allergy and Infectious Diseases, National Institutes of Health, Hamilton, Montana, United States of America; 2 *Salmonella*-Host Cell Interactions Section, Laboratory of Intracellular Parasites, Rocky Mountain Laboratories, National Institute of Allergy and Infectious Diseases, National Institutes of Health, Hamilton, Montana, United States of America; 3 Microscopy Unit, Research Technology Branch, Rocky Mountain Laboratories, National Institute of Allergy and Infectious Diseases, National Institutes of Health, Hamilton, Montana, United States of America; Washington University School of Medicine, United States of America

## Abstract

Reports implicating important roles for cholesterol and cholesterol-rich lipid rafts in host-pathogen interactions have largely employed sterol sequestering agents and biosynthesis inhibitors. Because the pleiotropic effects of these compounds can complicate experimental interpretation, we developed a new model system to investigate cholesterol requirements in pathogen infection utilizing DHCR24^−/−^ mouse embryonic fibroblasts (MEFs). DHCR24^−/−^ MEFs lack the Δ24 sterol reductase required for the final enzymatic step in cholesterol biosynthesis, and consequently accumulate desmosterol into cellular membranes. Defective lipid raft function by DHCR24^−/−^ MEFs adapted to growth in cholesterol-free medium was confirmed by showing deficient uptake of cholera-toxin B and impaired signaling by epidermal growth factor. Infection in the absence of cholesterol was then investigated for three intracellular bacterial pathogens: *Coxiella burnetii*, *Salmonella enterica* serovar Typhimurium, and *Chlamydia trachomatis*. Invasion by *S.* Typhimurium and *C. trachomatis* was unaltered in DHCR24^−/−^ MEFs. In contrast, *C. burnetii* entry was significantly decreased in −cholesterol MEFs, and also in +cholesterol MEFs when lipid raft-associated α_V_β_3_ integrin was blocked, suggesting a role for lipid rafts in *C. burnetii* uptake. Once internalized, all three pathogens established their respective vacuolar niches and replicated normally. However, the *C. burnetii*-occupied vacuole within DHCR24^−/−^ MEFs lacked the CD63-postive material and multilamellar membranes typical of vacuoles formed in wild type cells, indicating cholesterol functions in trafficking of multivesicular bodies to the pathogen vacuole. These data demonstrate that cholesterol is not essential for invasion and intracellular replication by *S.* Typhimurium and *C. trachomatis*, but plays a role in *C. burnetii*-host cell interactions.

## Author Summary

Clustered receptors associated with cholesterol-rich microdomains, termed lipid rafts, are thought to provide plasma membrane signaling platforms that bacterial pathogens can subvert to gain entry into host cells. Moreover, cholesterol has been implicated as a critical structural lipid of several pathogen-occupied vacuoles. Cumulative data supporting these models have principally been derived using inhibitors of cholesterol metabolism and various sterol sequestering compounds, agents that can lack specificity and cause unwanted cellular affects. Here, we employed a new system to investigate pathogen reliance on cholesterol for host cell colonization that utilizes mouse embryonic fibroblasts that can synthesize precursor sterols, but not cholesterol. Cells lacking cholesterol displayed strong defects in lipid raft-based signaling. However, no defects were observed in entry, vacuole development, and growth of *Salmonella enterica* and *Chlamydia trachomatis*, bacterial pathogens previously shown to rely on cholesterol for optimal host cell parasitism. Entry by *Coxiella burnetii*, the bacterial cause of human Q fever, was significantly decreased in cholesterol-negative cells as was trafficking of membranous material to the pathogen vacuole. However, subsequent bacterial replication was unaltered. Our results should prompt a reevaluation of the overall importance of cholesterol in bacterial pathogenesis with the described experimental system providing an alternative approach for such studies.

## Introduction

Cholesterol is essential for proper membrane structure and function in eukaryotic cells. In the plasma membrane, the high cholesterol content (20–25% of total plasma membrane lipid) significantly influences membrane fluidity and permeability [1], [2]. In addition, cholesterol-rich membrane microdomains, or “lipid rafts”, sequester proteins involved in signal transduction, membrane fusion, and phagocytosis [3]. Cholesterol also regulates endosomal trafficking [4]–[6] and serves as a precursor for steroids and vitamins [7]. Defects in cholesterol biosynthesis and/or trafficking can have severe consequences, including increased susceptibility to oxidative stress and apoptosis [8]–[10] as well as impaired lipid raft signaling and altered caveolae structure [11]–[13]. At the organismal level, defects in cholesterol trafficking and storage can lead to neurodegeneration and splenomegaly, while a complete lack of cholesterol during fetal development is fatal [14].

Mammalian cells have two primary ways of obtaining cholesterol: uptake of exogenous cholesterol through low density lipoprotein (LDL) and biosynthesis of endogenous cholesterol. *De novo* cholesterol synthesis occurs in the endoplasmic reticulum where the first sterol intermediate, lanosterol, is further modified by 19 enzymatic reactions of demethylation, hydroxylation, and double bond reduction to generate the final sterol product, cholesterol. At the terminal step, the carbon 24 double bond of desmosterol is reduced by a Δ24 sterol reductase. In the absence of this enzyme, membrane cholesterol is replaced by its precursor, desmosterol. The mammalian Δ24 sterol reductase, DHCR24/Seladin, is a bifunctional protein with an enzymatic role in cholesterol biosynthesis and a non-enzymatic role in conferring resistance to oxidative stress [10], [15], [16].

Cholesterol is considered a critical factor in host cell colonization by several bacterial pathogens. To gain entry into host cells, many bacteria target proteins enriched in plasma membrane lipids rafts including α_V_β_3_ integrin [17], E-cadherin [18], and ganglioside GM1 [19]. Furthermore, depletion of plasma membrane cholesterol with methyl-ß-cyclodextrin limits secretion of type III effector proteins by *Salmonella enterica* serovar Typhimurium and *Shigella flexneri*, resulting in decreased host cell invasion [20]. Filipin labeling shows high sterol levels in the membrane of intracellular compartments harboring pathogens such as *Coxiella burnetii* [21], *Chlamydia trachomatis* [22], and *S.* Typhimurium [23], leading to the hypothesis that cholesterol is critical for biogenesis of the pathogen-occupied vacuole. Another intracellular bacterium, *Anaplasma phagocytophilum*, recruits host cell cholesterol as a cell envelope constituent [24]. Cholesterol potentiates *A. phagocytophilum* infection of HL-60 cells [25] with trafficking of the sterol to the pathogen-occupied vacuole involving both LDL uptake and Niemann-Pick Type C pathways [25], [26]. *In vivo*, cholesterol promotes *A. phagocytophilum* infection of apolipoprotein E-deficient mice [27]. Pharmacological reagents that block LDL uptake dramatically inhibit *A. phagocytophilum* vacuole development and replication [25], while similar events are observed with *C. burnetii* and *C. trachomatis* infection when either cholesterol uptake or biosynthesis pathways are blocked [21], [22].

Commonly used cholesterol biosynthesis inhibitors and sequestering agents have pleotropic effects that can obscure the exact roles of cholesterol in host-pathogen interactions. For example, U18666A inhibits both trafficking of LDL [28], [29] and *de novo* cholesterol synthesis [30]. In addition, *de novo* synthesis inhibitors typically target cholesterol synthesis immediately upstream or downstream of lanosterol, therefore blocking synthesis of both intermediate sterols and cholesterol. Cholesterol-depleting compounds, such as methyl-ß-cyclodextrin, are toxic and significantly alter membrane properties such as protein diffusion and fluidity [31], [32]. Cells treated with methyl-ß-cyclodextrin also quickly replenish cholesterol-depleted membranes, thereby limiting experimental design. Collectively, these effects make defining a precise role for cholesterol in host-pathogen interactions challenging.

To circumvent the off-target effects of cholesterol biosynthesis inhibitors and sequestering agents, we established cholesterol-free cells using DHCR24^−/−^ mouse embryonic fibroblasts (MEFs) [10]. Using this system, we examined the ability of the bacterial pathogens *C. burnetii*, *S.* Typhimurium, and *C. trachomatis* to colonize cells in the absence of cholesterol. Surprisingly, and in contrast to previous reports, we found that cholesterol was not required for efficient invasion and growth of *C. trachomatis* and *S.* Typhimurium. However, our experiments revealed a role for cholesterol in *C. burnetii* host cell entry as well as trafficking to the pathogen vacuole.

## Results

### Culture conditions supporting growth of cholesterol-free DHCR24^−/−^ fibroblasts

**Figure 1:**
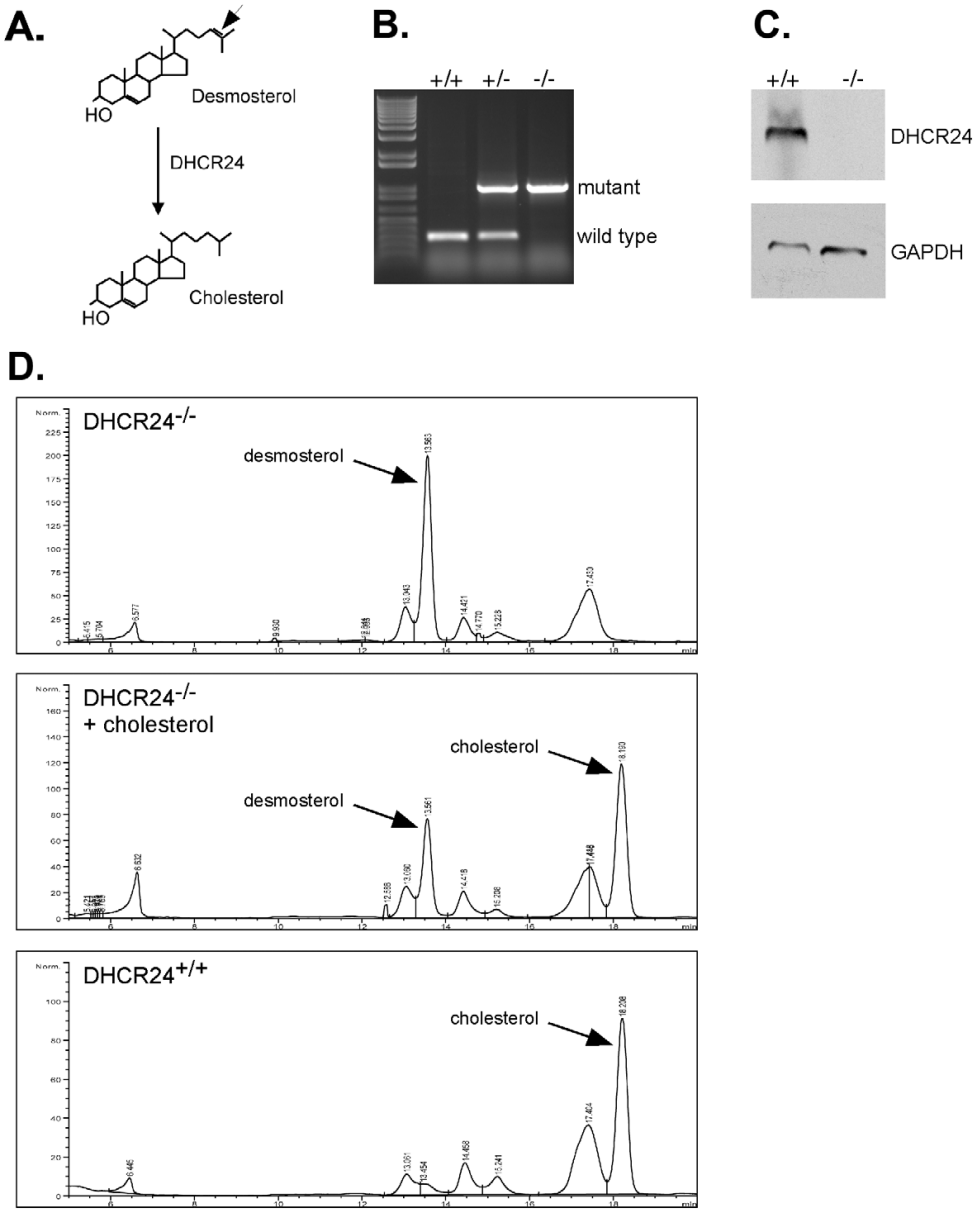
DHCR24^−/−^ cells adapted to serum-free media are cholesterol free. (A) The mammalian enzyme DHCR24 catalyzes the reduction of a double bond at carbon 24 (arrow) of desmosterol to generate cholesterol. DHCR24^−/−^ mouse embryonic fibroblasts (MEFs) were isolated and analyzed by PCR genotyping (B) and immunoblotting (C) to confirm the absence of DHCR24. The mutant and wild type alleles are indicated. GAPDH was probed as a loading control. (D) Sterol analysis by high pressure liquid chromatography (HPLC) demonstrated the absence of cholesterol in DHCR24^−/−^ MEFs once adapted to serum-free media (top panel). Desmosterol replaces cholesterol as the major sterol in these cells. The addition of exogenous cholesterol to DHCR24^−/−^ MEFs (middle panel) resulted in a sterol profile comparable to DHCR24^+/+^ wild type MEFs adapted to serum-free media (lower panel). Commercially available standards were used to determine the retention times of desmosterol and cholesterol.

The mammalian enzyme DHCR24 catalyzes the final step in cholesterol biosynthesis by reducing a double bond at carbon 24 [33] (Figure. 1A). In the absence of this enzyme, desmosterol, the immediate precursor of cholesterol, becomes the dominant sterol in cellular membranes. We hypothesized that DHCR24^−/−^ cells would provide a stable, cholesterol-free tissue culture system to study host-pathogen interactions. MEFs were isolated from a mating of heterozygote DHCR24^+/−^ mice and identified as DHCR24^−/−^ MEF lines by polymerase chain reaction (PCR) genotyping (Figure 1B). The absence of DHCR24 protein was confirmed by immunoblotting (Figure 1C). Although DHCR24^−/−^ MEFs cannot synthesize cholesterol, cultivation of cells in standard culture media with serum provides a rich source of exogenous cholesterol. To obtain cholesterol-free cells with no source of endogenous or exogenous cholesterol, DHCR24^−/−^ MEFs were adapted to medium lacking serum but containing the necessary primary fibroblast growth factors. Sterol analysis by high pressure liquid chromatography (HPLC) confirmed the absence of cholesterol in DHCR24^−/−^ MEFs adapted to serum-free media (referred to as −cholesterol MEFs) (Figure 1D, top panel), with desmosterol now present as the primary sterol. When DHCR24^−/−^ MEFs were grown in media supplemented with cholesterol (+cholesterol MEFs), cholesterol was preferentially incorporated into cellular membranes (Figure 1D, middle panel). As expected, cholesterol was the dominant sterol in wild type DHCR24^+/+^ MEFs even after adaptation to serum-free media (Figure 1D, bottom panel).

### Cholesterol-free MEFs have defective lipid raft function

**Figure 2:**
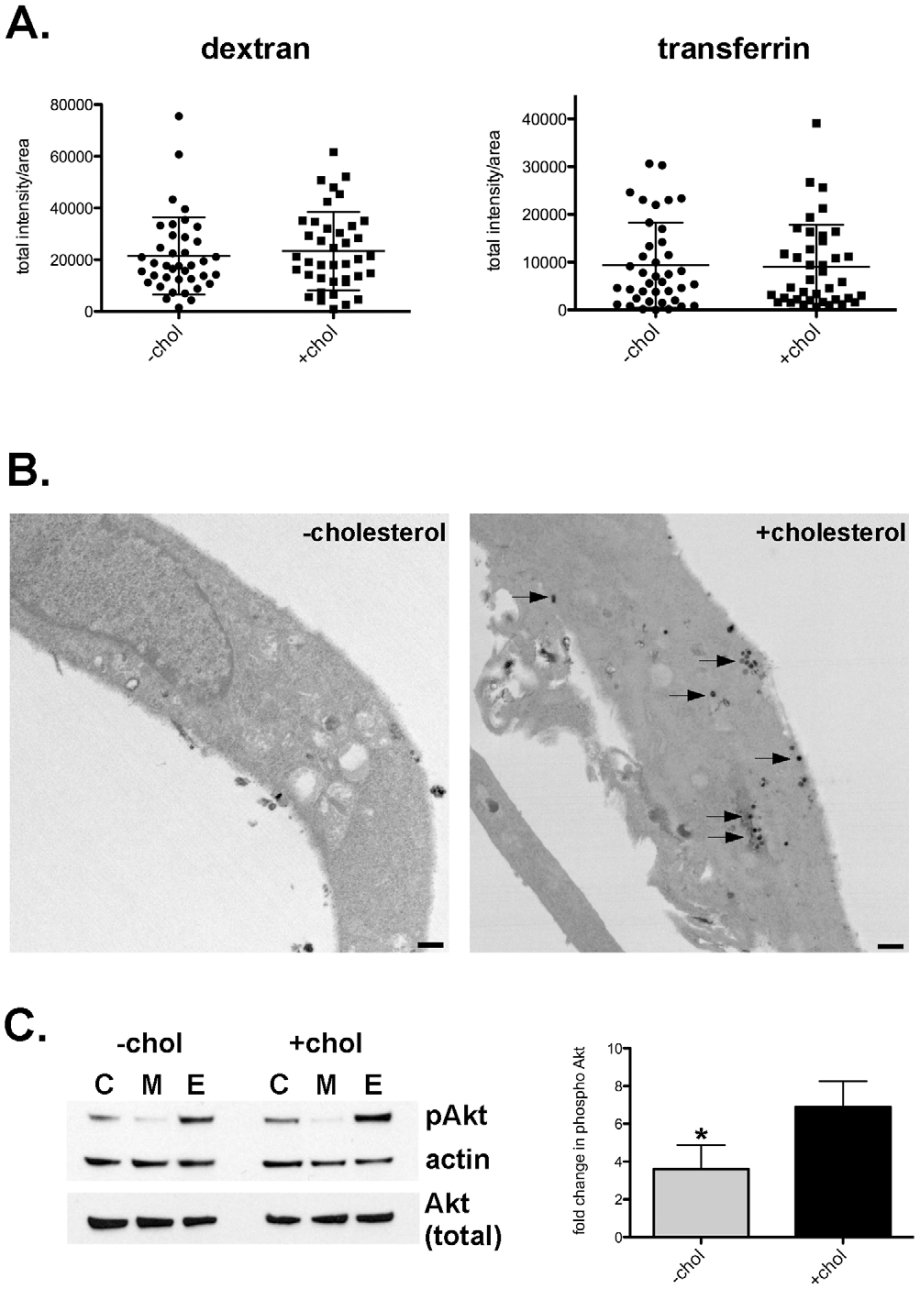
Lipid raft function is compromised in −cholesterol MEFs. (A). Scatter plot of semi-quantitative measurement of fluorescent dextran (fluid-phase) and transferrin (non lipid-raft) uptake. Each point represents the mean fluorescence intensity of an individual cell. Measurements for two independent experiments are shown with the mean and standard deviation indicated. No significant difference was found between −cholesterol and +cholesterol MEFs. (B). Representative transmission electron micrographs showing deficient uptake of cholera toxin-B (CT-B) in −cholesterol MEFs. Horseradish peroxidase (HRP)-labeled CT-B was added to cells and visualized by immunohistochemistry through the conversion of diaminobenzidine by HRP. In −cholesterol MEFs, very little to no CT-B-positive endosomes were observed, in contrast to +cholesterol MEFs (arrows). Scale bar = 500 nm. (C) Phosphorylation of Akt. (Left panel) Representative immunoblot of MEF lysates probed for Akt phosphorylated at Ser473, total Akt, and ß-actin as a loading control. C, control MEFs; M, serum starved MEFs; E, serum starved MEFs treated with epidermal growth factor (EGF) for 2 min. (Right panel) Densitometry of immuoblot showing that EGF stimulation resulted in a 6.9+/−0.73 fold increase in Akt (Ser473) phosphorylation by +cholesterol MEFs, but only a 3.6+/−0.78 fold increase by −cholesterol MEFs (p = 0.0373), indicating signaling through lipid rafts is impaired in −cholesterol MEFs. The mean +/− SD of three independent experiments is shown.

Desmosterol can replace cholesterol in tissue culture cells without major effects on growth and morphology [11], [34], [35]. However, in the absence of cholesterol, cells have impaired lipid raft function [10], [12]. To examine lipid raft function in −cholesterol MEFs, we first examined uptake of substrates through different endocytic processes. The fluid-phase marker dextran is internalized by cells through pinocytosis, a non-receptor mediated form of endocytosis. In contrast, receptor-mediated endocytosis can be either lipid raft-dependent (*e.g.*, cholera-toxin B) or -independent (*e.g*., transferrin). Internalization of dextran and transferrin, molecules that are both internalized independent of lipid rafts, was identical between −cholesterol and +cholesterol MEFs (Figure 2A). However, uptake of cholera toxin-B (CT-B), a process that relies on toxin binding of ganglioside GM1 in lipid rafts [36], was dramatically impaired in −cholesterol MEFs. By electron microscopy, only 9% of −cholesterol MEFs contained 10 or more CT-B-positive endosomes, compared to 80% of +cholesterol MEFs (Figure 2B and Figure S1). These results suggested that lipid raft-mediated uptake is defective in −cholesterol MEFs while other endocytic pathways function normally.

We next determined if lipid raft-mediated signaling was impaired in −cholesterol MEFs by investigating epidermal growth factor (EGF) receptor-mediated signal transduction. Binding of EGF by EGF receptor in lipid rafts triggers receptor dimerization and autophosphorylation, which leads to activation of multiple signaling proteins, including Akt [37]–[39]. Immunoblotting and densitometry was used to measure phosphorylated Akt after EGF stimulation. There was a 50% reduction (p = 0.0373) in phosphorylated (activated) Akt in −cholesterol MEFs when compared to +cholesterol MEFs (Figure 2C). Collectively, our data indicate that lipid raft function is significantly impaired in −cholesterol MEFs.

### *Salmonella* Typhimurium and *Chlamydia trachomatis* invade normally in the absence of plasma membrane cholesterol

**Figure 3:**
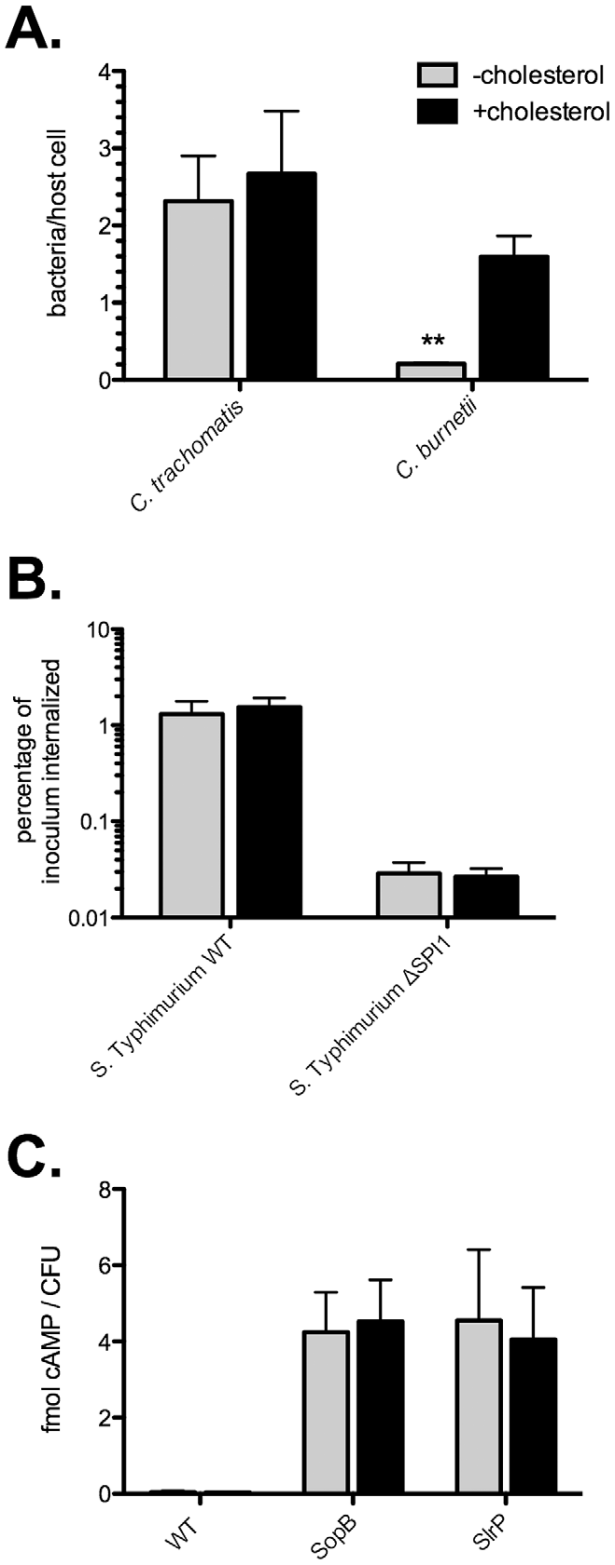
Entry of *C. burnetii*, but not *S.* Typhimurium or *C. trachomatis*, is reduced in the absence of cholesterol. Entry assays were performed to test the ability of *C. trachomatis*, *C. burnetii*, and *S.* Typhimurium to enter host cells in the absence of cholesterol. (A). The number of internalized *C. trachomatis* was unchanged between −cholesterol and +cholesterol MEFs. In contrast, internalization of *C. burnetii* was decreased by 87% (p = 0.0009) in −cholesterol MEFs. Error bars indicate the standard deviation from the mean of three independent experiments, each done in triplicate. (B). Wild type (WT) *S.* Typhimurium and a mutant lacking the *Salmonella* pathogenicity island 1 (ΔSPI1) invaded −cholesterol and +cholesterol cells with equal efficiency. (C). CyaA assay showing robust translocation of the *S.* Typhimurium type III effectors SopB and SlrP in both -cholesterol and +cholesterol MEFs. Normal cAMP levels were observed following infection with WT bacteria not expressing the fusion proteins (negative control). Error bars indicate the standard deviation from the mean of three independent experiments, each done in triplicate.

Studies using methyl-ß-cyclodextrin to deplete host cells of cholesterol suggest that cholesterol and/or cholesterol-rich lipid rafts facilitate entry of the intracellular bacteria *S.* Typhimurium and *Chlamydia trachomatis*, organisms that utilize a type III secretion system (T3SS) to actively induce their uptake [40]. The role of cholesterol in *C. burnetii* entry has not been explored; however, the pathogen enters passively through endocytic pathways [41], [42]. Recognizing the caveats of using cholesterol sequestering agents for pathogen uptake experiments, we conducted bacterial invasion assays using −cholesterol MEFs. We hypothesized that if lipid rafts are utilized by these pathogens to invade host cells, entry should be significantly decreased in −cholesterol MEFs. For *C. trachomatis* and *C. burnetii*, immunofluorescence staining was used to enumerate the average number of intracellular bacteria per cell, while a gentamicin protection assay was employed to measure the percentage of internalized *S*. Typhimurium. Interestingly, the infection efficiency for both *S.* Typhimurium and *C. trachomatis* was nearly identical between −cholesterol and +cholesterol MEFs (Figures 3A and 3B), indicating cholesterol is not required for host cell entry by these pathogens. The *Salmonella* pathogenicity island 1 (SPI1)-encoded T3SS was required for efficient bacterial internalization by both −cholesterol and +cholesterol MEFs, with a 50-fold decrease in invasion of a SPI mutant compared to wild type bacteria (Figure 3B). In contrast, uptake of *C. burnetii* by −cholesterol MEFs was decreased by 87% (p = 0.0009) when compared to +cholesterol MEFs (Figure 3A). This effect was not due to decreased *C. burnetii* adherence as pathogen attachment to -cholesterol or +cholesterol MEFs was similar (Figure S2).

### *S.* Typhimurium type III effector translocation does not require cholesterol

A study by Hayward *et al.* [20] demonstrated that depletion of cholesterol from NIH3T3 fibroblasts by treatment with methyl-ß-cyclodextrin inhibits translocation of the *S.* Typhimurium SPI1 effector proteins SopB and SopE. Furthermore, the type III secretion system 1 (T3SS1) translocon protein, SipB, was shown to bind cholesterol with high affinity using *in vitro* binding assays [20]. To further investigate the requirement of cholesterol in type III effector translocation, we used an adenylate cyclase (CyaA) assay to quantify translocation of two SPI1-associated effectors, SopB and SlrP, during *S.* Typhimurium infection of −cholesterol or +cholesterol MEFs. MEFs were infected with wild type *S.* Typhimurium expressing fusions of SopB or SlrP to *Bordetella pertussis* CyaA. At 1 hour post infection (hpi), cytosolic cAMP levels were measured by enzyme immunoassay and normalized to the number of bacterial colony forming units (CFU). As expected, −cholesterol and +cholesterol MEFs infected with wild type bacteria not expressing CyaA fusions showed normal levels of cAMP (Figure 3C; 0.046 and 0.033 fmol cAMP/CFU, respectively). In contrast, −cholesterol and +cholesterol MEFs infected with *S.* Typhimurium expressing either SopB-CyaA or SlrP-CyaA showed similar increases in levels of cAMP (Figure 3C; approximately 4 fmol cAMP/CFU), indicating translocation of effector fusions into the host cell cytoplasm. Thus, translocation of at least two *S.* Typhimurium T3SS1 effectors is independent of membrane cholesterol.

#### *C. burnetii* utilizes α_V_β_3_ integrin for entry into DHCR24^−/−^ fibroblasts

Previous studies have demonstrated that *C. burnetii* internalization by human monocytes involves α_V_β_3_ integrin [17]. Integrins are a family of 24 heterodimeric αβ transmembrane proteins that typically bind to extracellular matrix (ECM) proteins such as fibronectin, vitronectin, collagen, and laminin [43]. Binding of ECM proteins by integrins results in assembly of complex signaling networks that regulate many global responses. The α_V_β_3_ integrin is found in lipid rafts [44], and mediates cell motility [45], adhesion [46], and phagocytosis [47].

We were curious whether defective entry of *C. burnetii* into −cholesterol MEFs could be explained in part by deficient α_V_β_3_ integrin function. Expression of α_V_β_3_ integrin by −cholesterol and +cholesterol MEFs was first confirmed. By flow cytometry, we observed similar surface expression of α_V_β_3_ integrin by −cholesterol and +cholesterol MEFs (Figure S3). This result is consistent with published data showing that treatment of cells with cyclodextrin does not alter surface expression of raft-associated receptors [48], [49].

**Figure 4:**
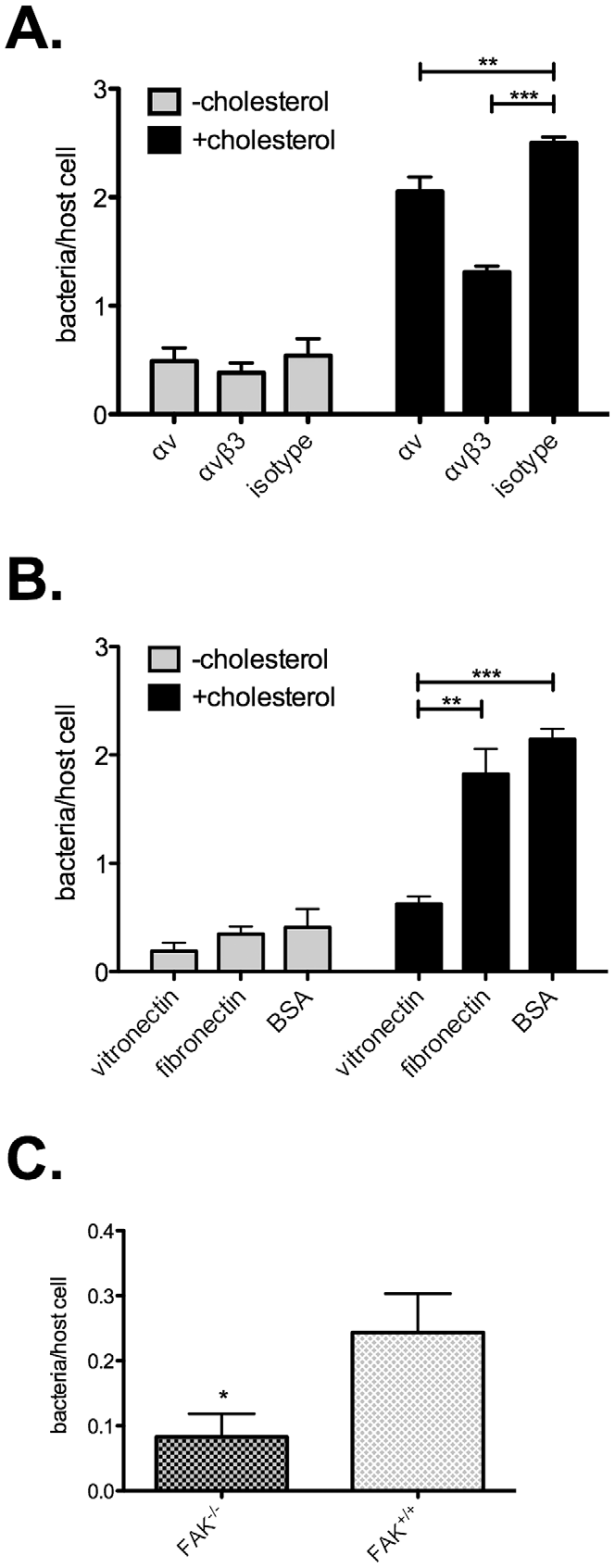
*C. burnetii* utilizes α_V_β_3_ integrin for uptake in a cholesterol-dependent manner. (A). Entry assays in the presence of α_V_β_3_ integrin blocking antibodies. *C. burnetii* internalization by −cholesterol MEFs was not significantly altered. In contrast, blocking either α_V_ integrin or α_V_β_3_ integrin in +cholesterol MEFs blocked *C. burnetii* entry by 18% (p = 0.0061) and 48% (p<0.0001), respectively. (B). When +cholesterol cells were pre-incubated with vitronectin, the major ligand for a α_V_β_3_ integrin, *C. burnetii* entry decreased by 66% (p = 0.001) and 71% (p<0.0001) as compared to pre-incubation with fibronectin or BSA, respectively. No effect was seen on −cholesterol MEFs. (C). Cells lacking the downstream α_V_β_3_ integrin signaling protein focal adhesion kinase (FAK) were tested for *C. burnetii* entry. As compared to wild type FAK^+/+^ cells, internalization decreased by 66% (p = 0.0160) in FAK^−/−^ cells. Error bars indicate the standard deviation from the mean of three independent experiments, done in triplicate.

The role of α_V_β_3_ integrin in *C. burnetii* entry was then examined by conducting uptake experiments in the presence of α_V_β_3_ integrin blocking antibodies and the α_V_β_3_ integrin ligand, vitronectin. In +cholesterol MEFs, blocking α_V_ integrin alone decreased *C. burnetii* entry by 18% (p = 0.0061), while blocking the α_V_β_3_ integrin heterodimer resulted in a 48% decrease in entry (p<0.0001) as compared to the isotype control (Figure 4A). Vitronectin is the primary ECM protein ligand for α_V_β_3_ integrin [50]. *C. burnetii* uptake decreased by 71% (p<0.0001) in +cholesterol MEFs pre-incubated with vitronectin, while pre-incubation with fibronectin or bovine serum albumin had no effect (Figure 4B). Neither blocking antibodies nor vitronectin altered entry in −cholesterol MEFs.

Upon ligand binding, α_V_β_3_ integrin triggers a cascade of downstream signaling events, including activation of focal adhesion kinase (FAK), a key regulator of integrin signaling. The involvement of α_V_β_3_ integrin in *C. burnetii* entry suggested that downstream proteins such as FAK would also be critical for *C. burnetii* uptake. To test this hypothesis, the ability of *C. burnetii* to colonize Du3 MEFs, a FAK^−/−^ cell line [51], [52], was assessed. Relative to wild type Du17 MEFs, infection of Du3 FAK^−/−^ MEFs resulted in a 66% decrease in entry (p = 0.0160) (Figure 4C). Together, these data suggest that *C. burnetii* utilizes the lipid-raft associated α_V_β_3_ integrin and its downstream signaling partners to gain entry into fibroblasts, and that defective signaling by this pathway in −cholesterol MEFs contributes to deficient uptake of *C. burnetii*.

### Intracellular replication is independent of cholesterol

**Figure 5:**
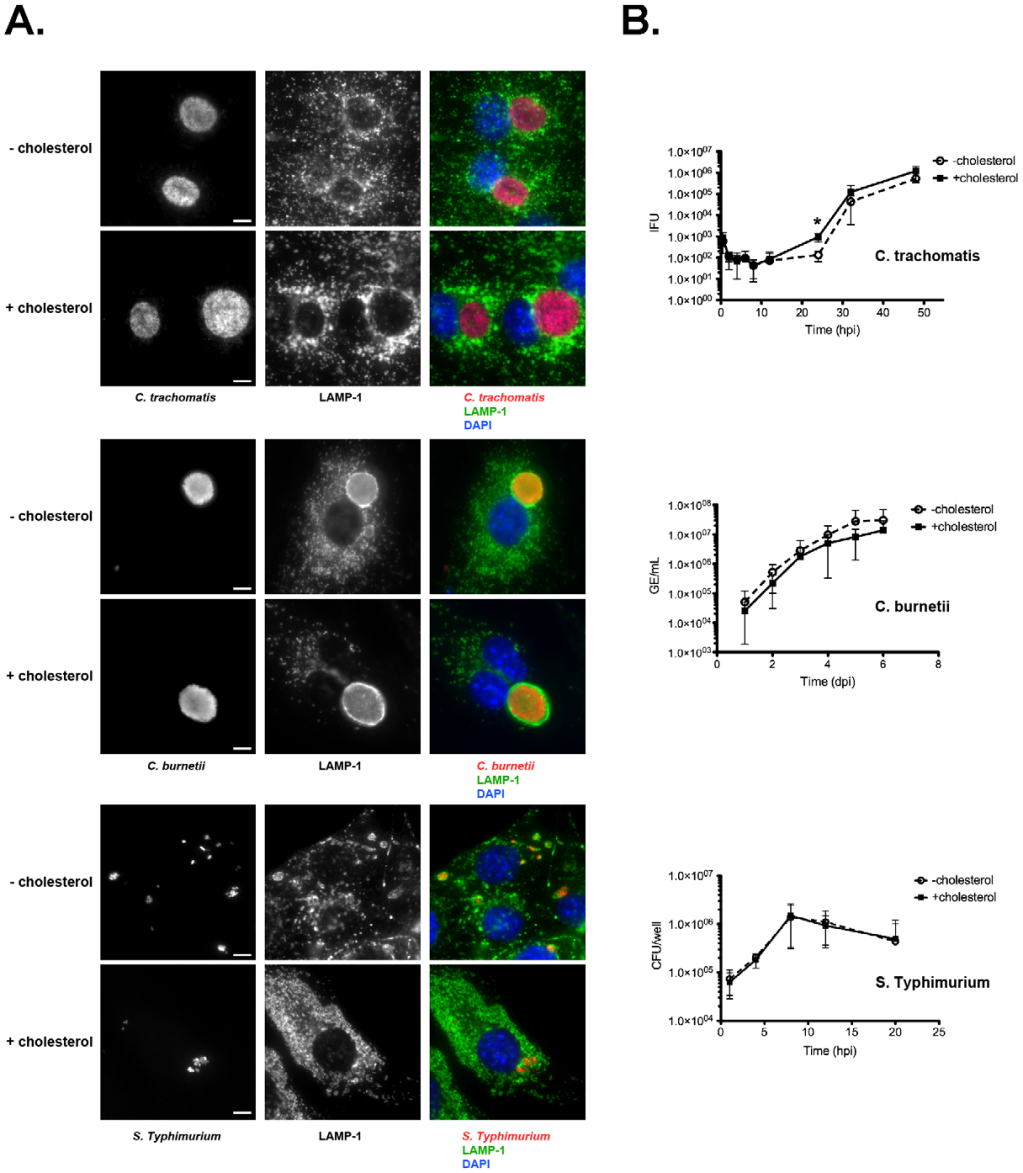
Pathogen growth in the absence of cholesterol is unaffected. (A). Fluorescence micrographs of infected −cholesterol and +cholesterol MEFs at 32 hpi (*C. trachomatis*), 4 dpi (*C. burnetii*), or 8 hpi (*S.* Typhimurium). Infected cells are stained by immunofluorescence for LAMP-1 (green), bacteria-specific antibodies (red), and DNA (blue). In all cases, there were no discernable differences in LAMP-1 staining or vacuole morphology. Scale bars = 5 µm. (B). Pathogen growth in −cholesterol and +cholesterol MEFs was measured by enumerating inclusion forming units (IFU; *C. trachomatis*), genome equivalents (GE; *C. burnetii*), or colony forming units (CFU; *S.* Typhimurium). Overall growth of all three pathogens in −cholesterol or +cholesterol MEFs was similar. However, the start of *C. trachomatis* logarithmic growth phase in −cholesterol MEFs was delayed, with 7-fold fewer infectious elementary bodies at the 24 h time point (p = 0.0272). Error bars indicate the standard deviation from the mean of three independent experiments done in triplicate.

To determine whether internalized *C. burnetii*, *S.* Typhimurium, and *C. trachomatis* can productively infect cells in the absence of cholesterol, we examined pathogen vacuole formation and replication during infection of −cholesterol and +cholesterol MEFs. MEFs were fixed and labeled by immunofluorescence at times post-infection when mature pathogen vacuoles are present. For all three bacteria, no notable difference in vacuole size or morphology was observed between −cholesterol and +cholesterol MEFs (Figure 5A). Furthermore, growth assays revealed no significant differences in overall bacterial replication (Figure 5B). However, development of infectious *C. trachomatis* elementary bodies (EB) from non-infectious reticulate bodies (RB) appeared delayed in −cholesterol MEFs, with fewer infectious *C. trachomatis* present at 24 hpi (p = 0.02).

### Cholesterol-dependent trafficking to the *C. burnetii* vacuole

**Figure 6:**
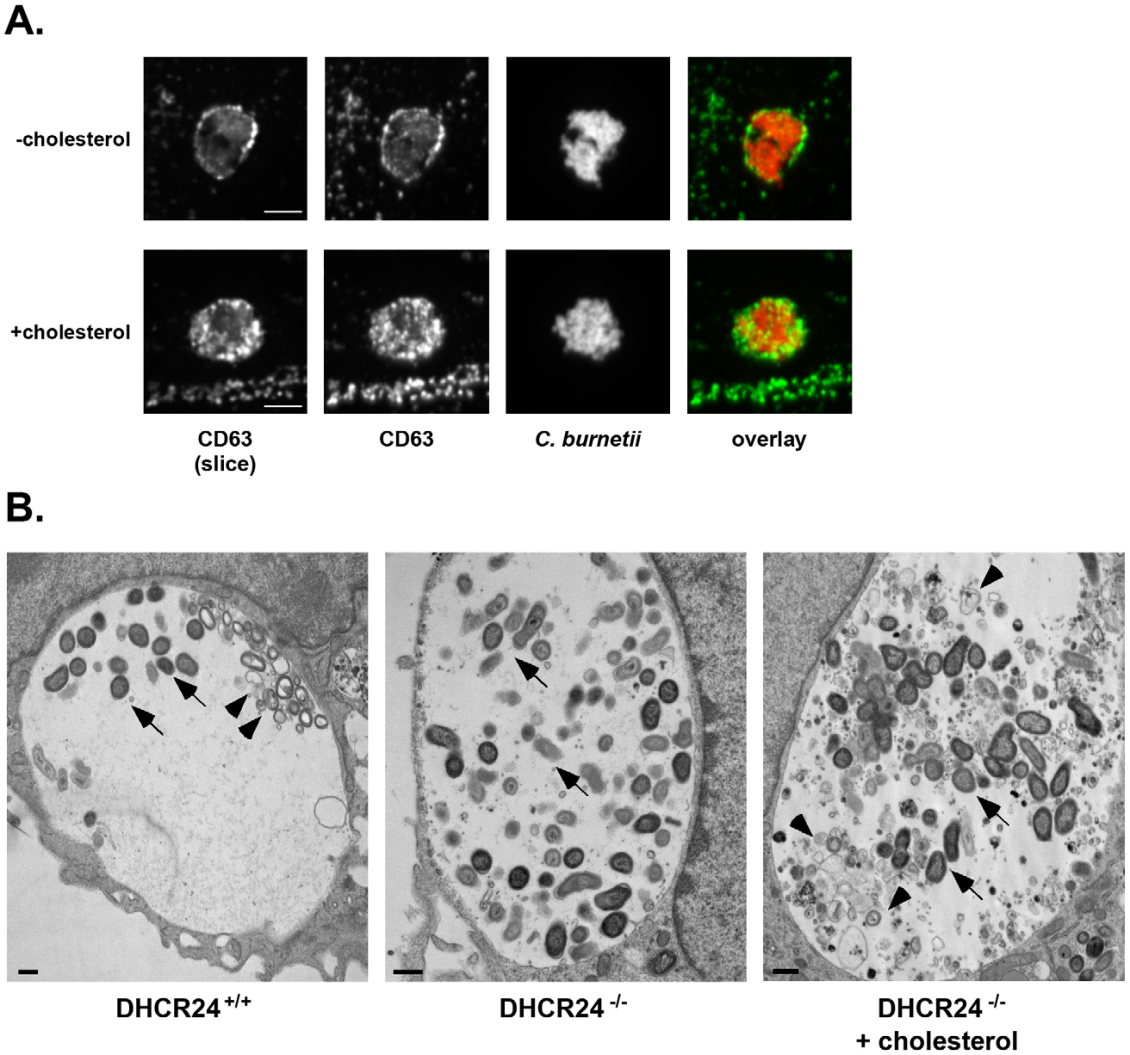
The lumenal contents of the *C. burnetii* vacuole are altered in the absence of cholesterol. (A). Fluorescence micrographs of *C. burnetii*-infected −cholesterol and +cholesterol MEFs stained for the late endosome/multivesicular body marker CD63 (green) and *C. burnetii* (red) at 4 dpi. Unlike +cholesterol MEFs, CD63 was not found in the lumen of vacuoles within −cholesterol MEFs. The first panel shows a single confocal slice through the middle of the vacuole, while the second panel is a maximum intensity Z projection of the entire vacuole. Scale bars = 5 µm. (B). Transmission electron microscopy revealed multilamellar membranous structures (arrowheads) within vacuoles harboring *C. burnetii* (arrows) in infected DHCR24^+/+^ and DHCR24^−/−^ +cholesterol MEFs, but not DHCR24^−/−^ −cholesterol MEFs. Scale bars = 500 nm.

Given the defective uptake of *C. burnetii* by −cholesterol MEFs (Figure 3A), we further characterized the *C. burnetii*-containing vacuole, which normally has characteristics of a large phagolysosome [53]. Vacuoles were immunostained for the endolysosomal markers CD63, Rab7, flotillin-2, syntaxin 7, syntaxin 8, or vamp7, and assayed for the presence of active cathepsin. With the exception of CD63, endolysosomal markers were associated with *C. burnetii* vacuoles regardless of the presence or absence of host cell cholesterol (data not shown). CD63 is a late endosome/multivesicular body marker [54] and is typically associated with both the *C. burnetii* vacuolar membrane and lumen [55]. However, CD63 was absent from the vacuole lumen in −cholesterol MEFs (Figure 6A). This observation correlated with ultrastructural differences in the contents of the *C. burnetii* vacuole (Figure 6B). In DHCR24^+/+^ wild type MEFs and DHCR24^−/−^ mutant MEFs with cholesterol added back, and as noted in other cell lines [56], a large amount of non-bacterial material was observed in the *C. burnetii* vacuole lumen, including multi-lamellar membranes and vesicles (Figure 6B). However, in DHCR24^−/−^ MEFs without cholesterol, there was a striking absence of this material (Figure 6B). Although the source and function of these membranous structures are unknown, we can speculate that at least some of the material is derived from multivesicular bodies (MVBs), based on the CD63 labeling of *C. burnetii* vacuole lumen. Together, these data suggest that trafficking of late endosomes/MVBs to the *C. burnetii* vacuole requires cholesterol.

### *C. burnetii* does not synthesize cholesterol from host cell precursors

**Figure 7:**
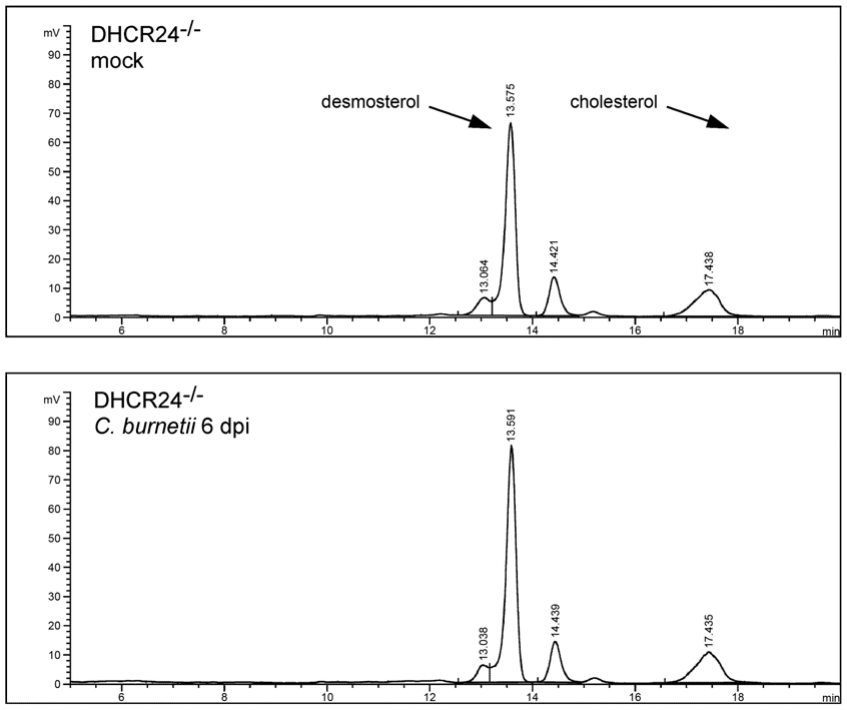
*C. burnetii* does not generate cholesterol in host cells. HPLC analysis of *C. burnetii*- and mock-infected −cholesterol MEFs at 6 dpi. Despite expressing a functional sterol reductase [59], there is no detectable cholesterol in infected cells, indicating *C. burnetii* does not generate cholesterol from host cell sterols.

*C. burnetii* is unique among bacteria in encoding two eukaryote-like sterol reductase homologs [57], [58]. One of these, CBU1206, is a functional Δ24 sterol reductase, as indicated by yeast complementation experiments [59]. The presence of this enzyme in *C. burnetii* suggested that the bacterium might synthesize cholesterol from mammalian sterol precursors, such as desmosterol. To test this hypothesis, we analyzed sterols from uninfected and infected −cholesterol MEFs by HPLC to determine if infected cells contained cholesterol. We found no evidence for cholesterol biosynthesis in *C. burnetii*-infected MEFs (Figure 7), which had a sterol profile virtually identical to uninfected cells. To confirm that cholesterol was not being made at very low levels, we labeled infected cells with ^14^C-acetate and analyzed the sterol profile by HPLC with an in-line scintillation detector. Even with this more sensitive detection method, there was no detectable cholesterol in *C. burnetii*-infected MEFs (data not shown).

## Discussion

Elucidating the role of cholesterol and lipid rafts in host-pathogen interactions has been challenging. The majority of studies have relied on cholesterol sequestering agents and biosynthesis inhibitors to remove or deplete host cell cholesterol. Cholesterol sequestering agents such as methyl-ß-cyclodextrin physically remove cholesterol from membranes, in particular the plasma membrane. While this method efficiently depletes cholesterol, several concerns must be recognized. First, standard treatment of cells with methyl-ß-cyclodextrin (5–10 mM) removes up to 90% of the total cellular cholesterol, resulting in dramatic effects on cellular morphology and viability [31]. Second, cells tightly regulate total cholesterol levels as well as concentrations across organelles. Removal of plasma membrane cholesterol can therefore alter cholesterol distribution throughout the cell and result in increased cholesterol synthesis and trafficking [60]. Finally, cyclodextrins remove cholesterol from both lipid raft and non-lipid raft domains, and there is some evidence that non-cholesterol membrane components such as phospholipids are also extracted [31]. As a result, membrane properties such as fluidity and protein distribution are drastically altered. Other agents used to interfere with cholesterol functions, such as filipin and nystatin, have similar negative effects on the host cell [61]. Another approach to cholesterol depletion utilizes biosynthesis inhibitors, such as statins and U18668. While inhibitors can efficiently lower cholesterol levels, the approach suffers from two major problems. First, the majority of available inhibitors target enzymes early in the pathway. Consequently, the resulting decrease in all sterols makes it difficult to directly associate results with cholesterol depletion. Second, the majority of the inhibitors have pleotropic and/or off-target effects. For example, U18666A inhibits trafficking of LDL-derived cholesterol [28], [29], *de novo* cholesterol synthesis [30], and lipid organization of membranes by directly binding to membranes [62].

When adapted to serum-free media, DHCR24^−/−^ MEFs lack cholesterol but contain all of the upstream sterols. In the place of cholesterol these cells accumulate desmosterol, a sterol that differs from cholesterol by a single double bond at the carbon 24 position. Previous work in J774 murine macrophages, which also lack the DHCR24 enzyme, demonstrates that desmosterol can replace cholesterol in the regulation of cellular sterol homeostasis and proliferation [35]. However, desmosterol does not functionally replace cholesterol in lipid rafts [10]–[12]. Indeed, we found lipid raft-mediated uptake and signaling to be dysfunctional in −cholesterol MEFs.

Many studies have examined the role of cholesterol and lipid rafts in pathogen-host cell interactions. However, to our knowledge, these studies have all utilized methyl-ß-cyclodextrin and/or non-specific inhibitors. Our cholesterol-free cell system allowed examination of the importance of cholesterol in host cell colonization of three intracellular bacterial pathogens (*C. burnetii*, *S.* Typhimurium, and *C. trachomatis*) without the pleiotropic effects of these pharmaceutical agents.

Invasion of host cells by the obligate intracellular bacterium *C. trachomatis* is promoted by cytosolic translocation of effector proteins via a T3SS [63]. However, conflicting reports exist on the role of cholesterol in the invasion process. Jutras and colleagues [64] found that *C. trachomatis* associates with detergent-resistant plasma membranes, and that bacterial uptake is decreased by 80% in cells treated with methyl-ß-cyclodextrin. In contrast, Gabel *et al.* [65] saw no effect on entry when cells were treated with methyl-ß-cyclodextrin, filipin, or nystatin. Our results agree with the latter report, as we saw no difference in *C. trachomatis* invasion of MEFs with or without cholesterol.

Once internalized, *C. trachomatis* resides in a vacuole (or inclusion) that disconnects from the endocytic pathway and acquires characteristics of a Golgi-derived vesicle [66]. Here, infectious EBs differentiate into metabolically active RBs. Several lines of evidence suggest a role for cholesterol in *C. trachomatis* inclusion development and replication. Filipin labeling indicates that both the inclusion membrane and bacteria contain sterols [22], [67], while HPLC analysis demonstrates the presence of cholesterol in the bacteria [22]. Furthermore, sterol delivery appears to be Golgi-dependent [22]. We found that cholesterol was not essential for productive infection by *C. trachomatis*, as the number of infectious units at 48 hpi was identical between MEFs with or without cholesterol. However, we did observe a delay in the onset of the logarithmic phase of the organism's growth cycle, suggesting cholesterol is involved in RB to EB transition. This may reflect a defect in trafficking to the inclusion, perhaps resulting in a decrease in nutrients important for *C. trachomatis* development.

Our data also demonstrate that cholesterol is not essential for type III secretion and productive infection by *S.* Typhimurium, a finding that directly contradicts previous reports [20], [68]. These disparate results may reflect different experimental approaches, with previous studies using methyl-ß-cyclodextrin for cholesterol depletion. As discussed earlier, this treatment alters the membrane properties of cells. Indeed, Garner *et al.* [68] observed that methyl-ß-cyclodextrin treated cells “exhibited a rounder morphology,” although they did not find a difference in cell viability. Hayward *et al.* [20] utilized *in vitro* binding assays to demonstrate binding of the *S.* Typhimurium T3SS protein SipB to cholesterol complexed with methyl-ß-cyclodextrin. However, other sterols, such as desmosterol, were not tested, nor was the ability of SipB to bind model membranes, a closer cellular mimic. By immunofluorescence, Hayward *et al.* [20] also showed defective secretion of the T3SS effector SopB in methyl-ß-cyclodextrin-treated cells. Using a more sensitive CyaA assay, we demonstrate here the cholesterol-independent translocation of the effectors SopB and SlrP.

Based on filipin staining, the *S.* Typhimurium vacuole accumulates cholesterol during host cell infection [23]. However, inhibitor studies suggest that non-sterol precursors of the cholesterol biosynthetic pathway are required for *S.* Typhimurium intracellular growth, and that cholesterol itself is not essential [69]. While these studies were not done in a truly cholesterol-free system (*i.e.*, normal serum conditions were used), our data support the conclusion that cholesterol is not essential for *S.* Typhimurium growth in host cells.

Unlike *C. trachomatis* and *S.* Typhimurium, *C. burnetii* passively enters host cells through receptor-ligand interactions, triggering classical actin-dependent phagocytosis [41], [42], [70]. *C. burnetii* entry into cholesterol-free cells is significantly decreased, suggesting uptake occurs through cholesterol or lipid raft-mediated pathway. Our data using blocking antibodies and vitronectin demonstrate that α_V_β_3_ integrin is involved in entry into MEFs in a cholesterol-dependent manner. Furthermore, FAK, a key component of integrin signaling, is required for efficient *C. burnetii* entry. Together, these data suggest that *C. burnetii* utilizes lipid raft-mediated α_V_β_3_ integrin signaling to gain entry into host cells. Although significantly decreased, *C. burnetii* entry into −cholesterol MEFs still occurs, suggesting the pathogen can also enter by non-lipid raft-associated receptors that function normally and/or by lipid raft-associated receptors that function inefficiently due to raft disruption.

Based on intense staining by the sterol-binding fluorophor filipin, we previously showed that the membrane of the mature *C. burnetii* vacuole is sterol-rich [21]. In the same study, inhibitors of host cell sterol biosynthesis and uptake inhibited *C. burnetii* vacuole formation and growth [21]. Here, we demonstrate that *C. burnetii* vacuole formation and replication in −cholesterol MEFs is similar to +cholesterol MEFs. We conclude from these data that precursors of cholesterol, but not cholesterol *per se*, are required for optimal infection by *C. burnetii*. The *C. burnetii*-occupied vacuole of −cholesterol MEFs does show a striking absence of CD63-positive membranous material that we speculate represents MVBs. Thus, trafficking to and fusion with the *C. burnetii* vacuole of some vesicular compartments appears to depend on cholesterol, although these events are clearly not required for pathogen replication.

The *C. burnetii* human DHCR24 homolog CBU1206 has sterol reductase activity when ectopically expressed in yeast [59]. Thus, we postulated that CBU1206 activity during *C. burnetii* infection of DHCR24^−/−^ MEFs might rescue the cholesterol-negative phenotype of these cells to result in enhanced pathogen growth. However, synthesis of cholesterol was not detected in infected cells; thus, the precise role of CBU1206 in *C. burnetii* colonization of mammalian cells remains unresolved.

To our knowledge, this is the first study to address the role of cholesterol in host-pathogen interactions without the use of pleiotropic inhibitors or compounds that dramatically change membrane dynamics. While our results argue that cholesterol is not absolutely required for *in vitro* host cell colonization by three different intracellular pathogens, it does not eliminate the possibility that these pathogens target cholesterol and lipid rafts during *in vivo* infection, or that cholesterol is important under specific cellular conditions.

## Materials and Methods

### Ethics statement

This study was carried out in strict accordance with the recommendations in the Guide for the Care and Use of Laboratory Animals of the National Institutes of Health. The animal protocol used in this study was approved by the Rocky Mountain Laboratories Institutional Animal Care and Use Committee (Protocol Number: RML 2008-08).

### Bacteria

*C. burnetii* Nine Mile RSA439 (phase II, clone 4) was propagated in African green monkey kidney (Vero) cells (CCL-81; ATCC, Manassas, VA) grown in RPMI with 10% fetal bovine serum (FBS) (Invitrogen, Carlsbad, CA). Bacteria were purified from host cells at 28 days post infection (dpi) as previously described [71], and stored at −80°C. *Chlamydia trachomatis* LGV-434, serotype L2, was propagated in HeLa 229 cells (CCL-2; ATCC) in RPMI +10% FBS. Bacteria were purified by Renografin density gradient centrifugation [72], and stored at −80°C. Wild type *Salmonella enterica* serovar Typhimurium (*S.* Typhimurium) SL1344 [73] and the isogenic ΔSPI1::kan mutant [74] have been previously described. *S.* Typhimurium bacteria were grown in 2 ml LB-Miller broth for 16 to 18 h at 37°C with aeration (225 rpm), then subcultured in 10 ml LB-Miller broth (1∶33 dilution) for 3.5 h (225 rpm). Prior to infection, bacteria were centrifuged at 8,000*xg* for 2 min and the bacterial pellet resuspended in an equal volume of Hank's Balanced Salt Solution (HBSS; Mediatech, Manassas, VA).

### Mammalian cell culture

Heterozygote DHCR24^+/−^ mice (C57BL/6 genetic background) were generously provided by Quark Biotech, Inc. (Ness Ziona, Israel). Individual 15 to 17 day embryos were harvested and digested with trypsin, and fibroblasts cultured in DMEM supplemented with 10% FBS and 100 U ml^−1^ penicillin/streptomycin (Invitrogen). DNA was isolated from both individual embryos and fibroblasts using a DNeasy Blood and Tissue DNA isolation kit (Qiagen, Valencia, CA). PCR genotyping was conducted as previously described [75].

To generate stable lines, MEFs were passaged every three days at 3.8×10^5^ cells per 25 cm^2^ flask in DMEM containing 10% FBS. Once stable lines were obtained (approximately passage 20), they were adapted to serum-free media using Fibroblast Basal Medium supplemented with compounds contained in a fibroblast serum-free growth kit (ATCC). MEFs were then continuously cultured in serum-free fibroblast media with or without SyntheChol, a water-soluble cholesterol media supplement (Sigma-Aldrich, St. Louis, MO) [76].

MEFs negative for focal adhesion kinase (Du3 cells; FAK^−/−^) and wild type cells (Du17 cells; FAK^+/+^) [51], [52] were grown in DMEM supplemented with 10% FBS.

### Immunoblotting

To determine DHCR24 protein levels, confluent MEF monolayers from a 25 cm^2^ flask were washed twice with phosphate-buffered saline (PBS; 1 mM KH_2_PO_4_, 155 mM NaCl, 3 mM Na_2_HPO_4_, pH 7.2), trypsinized, and pelleted at 1500*xg* for 5 min. Cell pellets were resuspended in sodium dodecyl sulfate (SDS) sample buffer (0.5% SDS, 50 mM Tris, 150 mM NaCl) and the protein concentration determined using a DC protein assay kit (Bio-Rad, Hercules, CA). Ten micrograms of protein per lane were separated by 10% SDS-polyacrylamide gel electrophoresis (SDS-PAGE) and transferred to nitrocellulose. The membrane was probed with a mouse polyclonal antibody directed against human DHCR24 (Abnova, Walnut, CA) for 1 h, followed by incubation with an anti-mouse immunoglobulin G secondary antibody conjugated to horseradish peroxidase (HRP) (ThermoScientific, Rockford, IL). Following development using chemiluminesence (ThermoScientific), the membrane was treated for 1 h with 0.1% azide in PBS to destroy the chemiluminescent signal, then reprobed for glyceraldehyde-3-phosphate dehydrogenase (GAPDH) as a loading control using rabbit anti-GAPDH (Cell Signaling, Danvers, MA) and anti-rabbit-HRP (ThermoScientific).

To analyze Akt phosphorylation, confluent MEF monolayers were starved in serum-free media without growth supplements for 3 h. Cells were stimulated with 50 ng ml^−1^ recombinant mouse epidermal growth factor (EGF; ATCC) for 2 min prior to lysis in SDS sample buffer as indicated. Proteins were separated on 4–20% SDS-PAGE gradient gels (Bio-Rad, Hercules, CA), then transferred to nitrocellulose. The membrane was probed with rabbit anti-phospho Ser473 Akt (Cell Signaling), total Akt (Cell Signaling), and mouse anti-ß actin (Abcam, Cambridge, MA). Densitometry was conducted using a Kodak Image Station 4000 MM (Eastman Kodak, Rochester NY), and phospho Akt levels normalized to actin. Phospho Akt signals were compared between EGF-stimulated MEFs and mock stimulated MEFs.

### Dextran and transferrin uptake

MEFs were scraped into fresh media, counted, and the cell density adjusted to 1×10^5^ cells ml^−1^. Resuspended cells were plated onto an ibidi-treated channel μ-slide VI^0.4^ (Ibidi, Verona, WI) and allowed to adhere. Prior to labeling, the ibidi dish was chilled on ice for 5 to 10 min. Transferrin Alexa Fluor 546 (50 µg ml^−1^, Invitrogen) or dextran Alexa Fluor 647 (1 mg ml^−1^, Invitrogen) was added in serum-free media, then the culture dish was incubated on ice for 15 min followed by incubation at 37°C for 15 min. The cells were washed twice with cold media, then treated with basal media (pH 3.5) to remove extracellular label. Cells were fixed for 15 min on ice with 2.5% paraformaldehyde (PFA) in PBS. Identical capture settings were used to obtain 0.4 µm slices with a modified Perkin-Elmer UltraView spinning-disk confocal system connected to a Nikon Eclipse Ti-E inverted microscope. ImageJ (written by W.S. Rasband, National Institutes of Health, Bethesda, MD) was used to quantitate intensity per cell area, with identical threshold settings. At least 20 cells were measured per condition for each experiment (n = 2).

### Determination of sterol content

Lipids were extracted using a modified Bligh-Dyer protocol [77]. Confluent monolayers from a 25 cm^2^ flask were washed twice with PBS, scraped into 10 ml PBS, and centrifuged for 5 min at 1500*xg*. The cell pellet was resuspended in 1.6 ml PBS and transferred to a glass tube. Chloroform:methanol 1∶2 (6 ml) was added and the solution vortexed. Following the addition of 2 ml chloroform and 2 ml PBS, the aqueous and organic phases were separated by centrifugation at 1500*xg* for 5 min. The lower organic phase was transferred to a new glass vial, dried under nitrogen, and resuspended in a small volume of acetonitrile:methanol (50∶50). Sterols were analyzed by HPLC using a C18 reverse phase column (Alltima HP C18 HL 5 µm, 250×4.6 mm, Grace Davison Discovery Sciences, Deerfield, IL) at 51°C, with a mobile phase of methanol:acetonitrile 50∶50 at 1 ml min^−1^. Sterol fractions were detected with an evaporative light scattering detector set at 60°C. The retention times of desmosterol and cholesterol were determined using commercially available standards (Avanti Polar Lipids, Alabaster, Alabama).

### Entry assays

Fluorescence-based in versus out assays were employed for *C. burnetii* and *C. trachomatis*. MEFs were scraped into fresh media, counted, and the concentration adjusted to 1.5×10^5^ cells ml^−1^. Resuspended cells were plated into individual channels of an ibidi-treated channel μ-slide VI^0.4^ (Ibidi, Verona, WI). *C. burnetii* and *C. trachomatis* were added to MEFs at a multiplicity of infection (MOI) of 200 (based on genome equivalents) and 10 (based on inclusion-forming units), respectively, and incubated at 37°C in 5% CO_2_ for 2 h (*C. burnetii*) or 1 h (*C. trachomatis*). Cells were then fixed with 2.5% PFA for 15 min on ice, followed by three washes with PBS. All subsequent steps were done at room temperature. Cells were blocked for 15 min in 1% bovine serum albumin (BSA) in PBS, then incubated for 15 min with rabbit polyclonal antibody directed against *C. burnetii* or a mouse monoclonal antibody (L21-45) directed against *C. trachomatis* serovar L2. After six washes with PBS to remove any residual antibody, cells were permeabilized for 15 min with 0.1% Triton X-100 in 1% BSA-PBS, followed by incubation for 15 min with guinea pig polyclonal antibody directed against *C. burnetii* or rabbit polyclonal antibody directed against *C. trachomatis*. After five washes with PBS, cells were incubated with secondary antibodies for 15 min (for *C. burnetii*, Alexa Fluor 546 anti-rabbit and Alexa Fluor 488 anti-guinea pig; for *C. trachomatis*, Alexa Fluor 546 anti-mouse and Alexa Fluor 488 anti-rabbit). After washing five times with PBS, samples were mounted in ProLong Gold containing DAPI (4′,6-diamidino-2-phenylindole; Invitrogen). The number of intracellular (green only) bacteria per cell were counted. Experiments were conducted three times in triplicate, with 100 cells counted per replicate. The results are expressed as the total number of intracellular bacteria divided by the total number of host cells.

For blocking assays, cells were plated on ibidi-treated channel slides as described above. Prior to infection, cells were incubated with 120 µl of blocking antibody or protein at 10 µg ml^−1^ for one h. Mouse anti-α_V_ integrin (monoclonal 272-17E6), mouse anti- α_V_β_3_ integrin (monoclonal 27.1), mouse vitronectin and mouse fibronectin were obtained from Abcam (Cambridge, MA). Mouse IgG1 isotype control antibody was obtained from BD Biosciences (San Jose, CA). *C. burnetii* was added to MEFs at an MOI of 200 in media containing corresponding antibody or protein, and after a 2 h incubation, the slides were fixed and processed for in/out staining as described above. Experiments were conducted three times in duplicate.

For *S.* Typhimurium CFU-based invasion assays, MEFs were seeded at ∼50% confluency in 24-well tissue culture treated plates approximately 24 h prior to infection. Bacteria were added to cells at an MOI of ∼100 and incubated for 10 min at 37°C. Extracellular bacteria were removed by aspiration, monolayers washed three times with HBSS, then fresh growth media was added to cell cultures. Following a 20 min incubation, cells were treated with growth media containing 50 µg ml^−1^ gentamicin for 1 h to kill extracellular bacteria. For enumeration of intracellular bacteria, monolayers were lysed in 1 ml 0.2% (w/v) deoxycholate in PBS and serial dilutions were plated onto LB agar plates to determine CFU. Experiments were done three times in duplicate, and internalized bacteria expressed as a percentage of the inoculum.

### *C. burnetii* adherence assay

MEFs were plated on ibidi-treated channel μ-slide VI^0.4^ as described above. *C. burnetii* was added to cells at an MOI of 200 and incubated on ice for 30 min. Cells were then fixed with 4% PFA for 15 min on ice, followed by three PBS washes. All subsequent steps were done at room temperature. After blocking in 1% BSA-PBS for 15 min, bacteria were labeled for 15 min with guinea pig anti-*C. burnetii*. After 3 washes with PBS, cells were incubated for 15 min with Alexa Fluor 546 anti-guinea pig and Alexa Fluor 488 wheat germ agglutinin to label adherent bacteria and the host cell plasma membrane, respectively. After three washes with PBS, cells were mounted with ProLong Gold with DAPI, and the number of attached bacteria counted. Experiments were done twice in triplicate, and results are expressed as the total number of attached bacteria divided by the total number of cells.

### Adenylate cyclase assay

For measurement of type III effector translocation, wild type *S.* Typhimurium bacteria were electroporated with low-copy number plasmids expressing SopB-CyaA-SigE [78] or SlrP-CyaA under the control of their native promoters. Overlap extension PCR was used to create a plasmid encoding the N-terminal 207 amino acids of SlrP fused to the catalytic domain of CyaA and under the control of the *slrP* promoter. *slrP* and approximately 470 bp of upstream region were amplified from *S*. Typhimurium SL1344 genomic DNA with the oligonucleotide pairs slrP-Xba-Fw (5′ tgc tct aga gcg agt cat cgt tac cat ggc tcg 3′) and slrP-cya-Rv (5′ acc agc ctg atg cga ttg ctg atc gag tat cag agt agt tat ctg ctc 3′). The catalytic domain of *Bordetella pertussis* CyaA was amplified from pPipB(1-210)-CyaA [79] with the oligonucleotide pairs cya-SlrP-OE-Fw (5′ cag ata act act ctg ata ctc gat cag caa tcg cat cag gct ggt tac 3′) and cyaA-Eco (5′ ccc gga tcc gat atc ttc atc gat aac tgt cat agc cgg 3′). The resulting amplicons were purified and mixed for a second round of PCR with slrP-Xba-Fw and cyaA-Eco. This amplicon was then cloned into pCR2.1TOPO (Invitrogen), released by EcoRI digestion and ligated into EcoRI-digested pMPMA3ΔPlac [80] to create SlrP-CyaA.

MEFs were infected as described above with wild type *S.* Typhimurium expressing or not expressing the SopB-CyaA-SigE or SlrP-CyaA fusion proteins. At 1 hpi, MEFs were washed twice with HBSS, then lysed in 1 ml 0.2% DOC for CFU enumeration, or 300 µl lysis buffer 1B (supplied with the cAMP immunoassay kit, GE Life Sciences, Piscataway, NJ) supplemented with 0.1 M HCl for CyaA assays. CyaA samples were rocked at room temperature for 10 min, neutralized with 1 M NaOH, then stored at −20°C. Samples were clarified by centrifugation at 14,000*xg* for 10 min at 4°C, then CyaA assays conducted using the non-acetylation enzymatic immunoassay procedure as described by the manufacturer (GE Life Sciences). The concentration of cAMP was normalized to CFUs collected at 1 hpi.

### Growth assays and pathogen vacuole development

MEFs were plated at 5×10^4^ cells per well in a 24-well tissue culture plate and allowed to grow to confluency. MEFs were infected with *C. burnetii* at an MOI of 25 for 2 h in 250 µl serum-free media. MEFs were washed twice with PBS, then incubated for the indicated times in serum-free media with or without SyntheChol. At each timepoint, the media was transferred to a 2 ml microfuge tube, the cells trypsinized and combined with the media. Following centrifugation at 20,000*xg* for 10 min, the pellet was resuspended in 200 µl water and added to a half volume of 0.1 mm zirconia/silica beads (BioSpec Products, Bartlesville, OK). Cells were lysed by bead beating in a FastPrep FP120 (Thermo Scientific) at setting #5 for 40 sec, the lysate spun briefly, then the sample heated at 100°C for 10 min. Beads and cell debris were pelleted at 20,000*xg* for 2 min, then 100 µl of the supernatant transferred to a new tube. Genome equivalents using a *dotA* probe were determined by quantitative PCR as previously described [56]. Growth assays were done three times in duplicate.

An inclusion-forming unit assay was used to quantify *C. trachomatis* growth [81]. Bacteria were added to MEF cultures at an MOI of 10 in 250 µl serum-free media, then cell cultures incubated on ice for 15 min followed by a 30 min incubation at 37°C. MEFs were washed twice with PBS, then fresh serum-free media with or without SyntheChol was added to the wells. At the indicated time points, the media was removed and the cells lysed by treatment with 500 µl of water for 5 min. After vigorous mixing, 10-fold serial dilutions were made in RPMI with 10% FBS. Bacterial suspensions were added to confluent HeLa cell monolayers that were then incubated at 37°C in 5% CO_2_ for 24 to 36 h. Infected HeLa cells were fixed with methanol, then inclusions stained with rabbit anti-*C. trachomatis* L2 antibody and anti-rabbit Alexa Fluor-488 secondary antibody. The number of inclusions in 15 fields at 20× were counted, and the experiment was done three times in duplicate.

To quantify *S.* Typhimurium intracellular replication, MEFs were infected as described for entry assays. After the initial 50 µg ml^−1^ gentamicin treatment, the gentamicin concentration was reduced to 10 µg ml−^1^ and replenished at 12 hpi when needed. CFU enumeration of intracellular bacteria was conducted as described above.

To assay pathogen vacuole development, infected MEFs in ibidi-treated channel μ-slide VI^0.4^ were fixed with 2.5% PFA, washed three times with PBS, then blocked/permeabilized for 15 min at room temperature with 0.1% saponin in 1% BSA-PBS. Primary antibodies used were rat anti-LAMP1 (BD Biosciences, San Jose, CA), guinea pig anti-*C. burnetii*, rat anti-CD63 (R&D Systems, Minneapolis, MN), and rabbit anti-*C. trachomatis*. Wild type *S.* Typhimurium constitutively expressing mCherry [82] was used for infections. Cells were mounted in ProLong Gold with DAPI. Widefield microscopy was done on Nikon Eclipse TE2000 epifluorescence microscope. Image analyses were done with ImageJ.

### Flow cytometry

MEFs were scraped into fresh media, spun at 500xg for 5 min, then washed once in cold 2% BSA-PBS. All subsequent manipulations were done on ice. Following a 10 min incubation in 2% BSA-PBS, mouse anti-α_V_β_3_ integrin (monoclonal 27.1) or mouse IgG1 isotype was added at a dilution of 1∶250. The cells were incubated for one h, washed twice with BSA-PBS, then incubated for 30 min with anti-mouse Alexa Fluor 647. Following four washes, the cells were analyzed by flow cytometry. Data were obtained on a LSR II flow cytometer (BD Biosciences) and analyzed using FlowJo version 8.3.3 (Tree Star, Inc., Ashland, OR).

### Transmission electron microscopy

Cells in 6-well plates were infected with *C. burnetii* for 4 days, washed twice with PBS, then fixed overnight in 2.5% glutaraldehyde/0.5 M sucrose in 0.1 M sodium cacodylate pH 6.8. Samples were processed and visualized with a model H7500 electron microscope (Hitachi High-Technologies USA, Pleasanton, CA) at 80 kV as previously described [83]. To visualize cholera toxin-B (CT-B) uptake, cells in 6-well plates were incubated with 1 ug ml^−1^ CT-B conjugated to HRP for 20 min on ice. After 3 washes with cold PBS, warm growth media was added and the cells incubated for 15 min at 37°C. Cells were washed an additional three times with cold PBS, then fixed with 2.5% glutaraldehyde/0.5 M sucrose in 0.1 M sodium cacodylate pH 6.8 for 1 h at room temperature before washing with 50 mM Tris-HCL (pH 7.4) plus 7.5% sucrose. HRP activity was detected by development with metal enhanced 3,3′-diaminobenzidine reagent (Thermo Scientific) for 1 h at room temperature prior to fixation and processing as described above. The number of HRP-positive endosomes in −cholesterol or +cholesterol MEFs were counted (n>50 cells), and cells scored as having more or fewer than 10 positive endosomes.

### Statistical analysis

Unless otherwise noted, results are presented as the mean of 3 independent experiments +/− S.D, and statistical significance determined by two-tailed unpaired student t test (Prism, GraphPad Software Inc, La Jolla, CA).

### Accession number

The sequence for CBU1206 is available in the NCBI database under accession number NC_002971.3.

## Supporting Information

Figure S1 **Electron microscopy analysis of cholera-toxin B uptake.** Bar graph showing enumeration of cholera-toxin B (CT-B)-positive endosomes in −cholesterol and +cholesterol MEFs. Results are expressed as the percentage of cells that had either fewer or more than 10 CT-B endosomes per cell.
(TIFF)

Figure S2 ***C. burnetii*** **attachment is not cholesterol-dependent.** The ability of *C. burnetii* to attach to DHCR24^−/−^ MEFs was determined using a fluorescence-based adherence assay. There was no significant difference in attachment of *C. burnetii* to −cholesterol or +cholesterol MEFs. Error bars indicate standard deviation from the mean of three independent experiments done in triplicate.
(TIFF)

Figure S3 **Expression of α_V_β_3_ integrin by DHCR24^−/−^ MEFs.** Flow cytometry of −cholesterol and +cholesterol MEFs reveals no significant difference in surface α_V_β_3_ integrin expression. The grey histogram depicts mouse IgG1 isotype control staining.
(TIF)
