# Supplementary figures and images for: Bacterial Colonization of Host Cells in the Absence of Cholesterol
Source: PLoS Pathog. 2013 Jan 24;9(1):e1003107. doi: 10.1371/journal.ppat.1003107 (PMC3554619; doi:10.1371/journal.ppat.1003107)

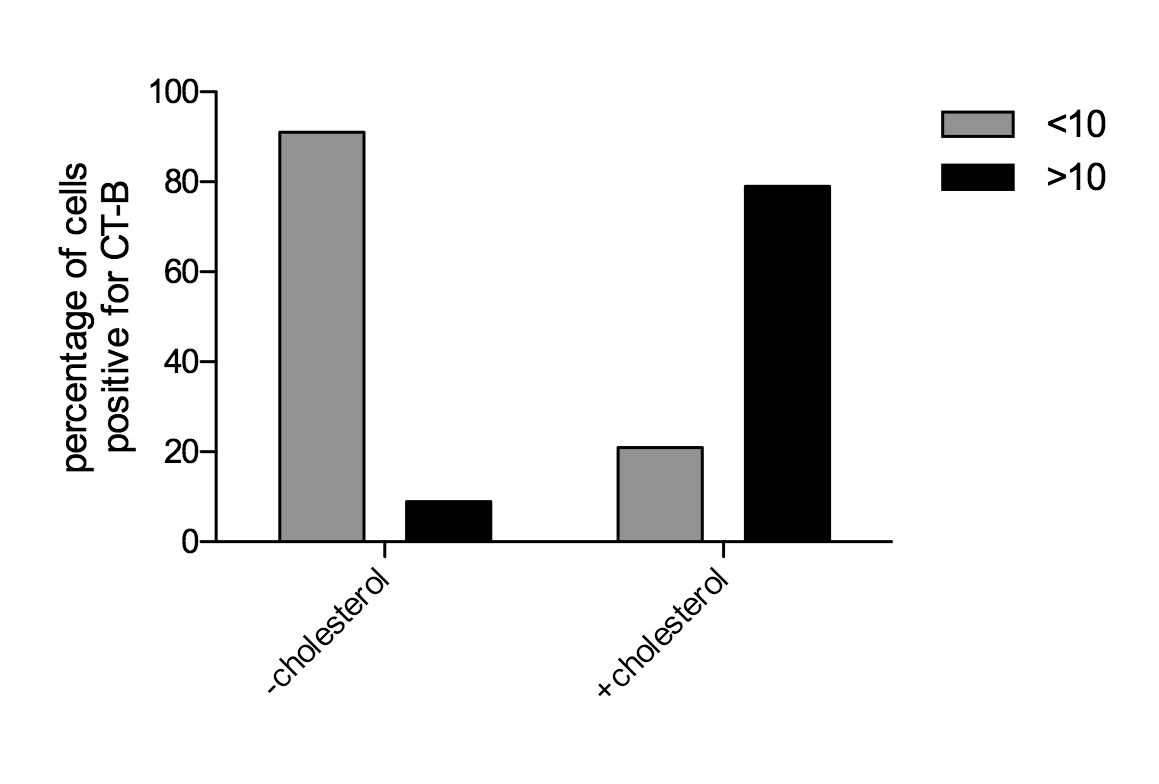

Supplement: Figure S1 — Electron microscopy analysis of cholera-toxin B uptake. Bar graph showing enumeration of cholera-toxin B (CT-B)-positive endosomes in −cholesterol and +cholesterol MEFs. Results are expressed as the percentage of cells that had either fewer or more than 10 CT-B endosomes per cell. (TIFF) [file ppat.1003107.s001.tiff]

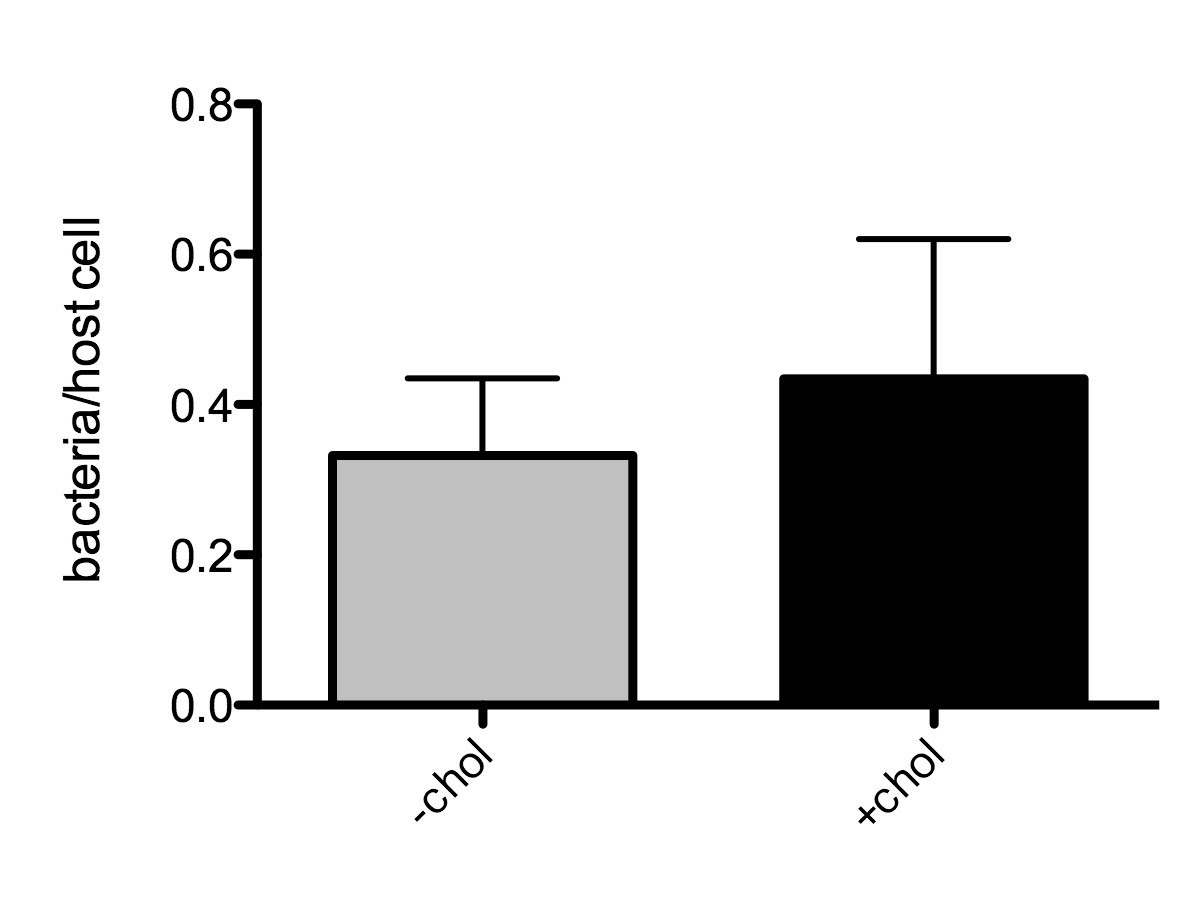

Supplement: Figure S2 — C. burnetii attachment is not cholesterol-dependent. The ability of C. burnetii to attach to DHCR24−/− MEFs was determined using a fluorescence-based adherence assay. There was no significant difference in attachment of C. burnetii to −cholesterol or +cholesterol MEFs. Error bars indicate standard deviation from the mean of three independent experiments done in triplicate. (TIFF) [file ppat.1003107.s002.tiff]

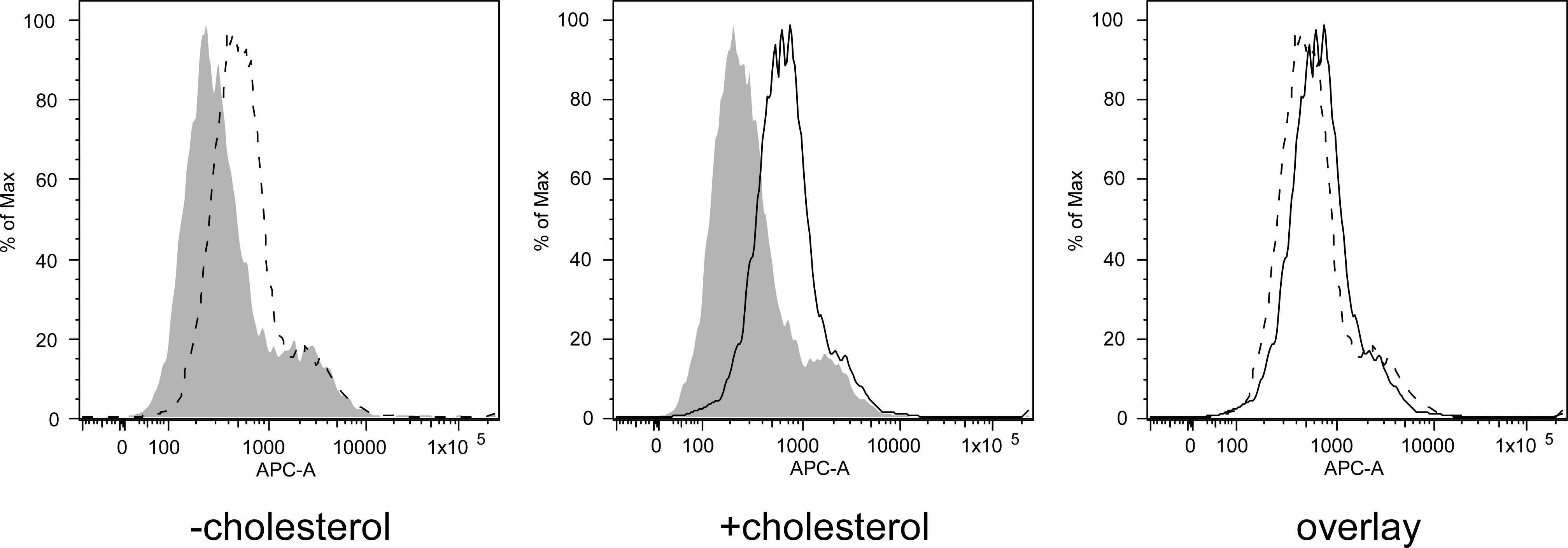

Supplement: Figure S3 — Expression of αVβ3 integrin by DHCR24−/− MEFs. Flow cytometry of −cholesterol and +cholesterol MEFs reveals no significant difference in surface αVβ3 integrin expression. The grey histogram depicts mouse IgG1 isotype control staining. (TIF) [file ppat.1003107.s003.tif]
